# Misconceptions about Ebola seriously affect the prevention efforts: KAP related to Ebola prevention and treatment in Kouroussa Prefecture, Guinea

**DOI:** 10.11694/pamj.supp.2015.22.1.6269

**Published:** 2015-10-10

**Authors:** Benti Geleta Buli, Landry Ndriko Mayigane, Julius Facki Oketta, Aguide Soumouk, Tamba Emile Sandouno, Bole Camara, Mory Saidou Toure, Aissata Conde

**Affiliations:** 1 African Union Support to Ebola Outbreak in West Africa (ASEOWA); 2 World Health Organization; 3 National Ebola Prevention and Control Coordination Body, Kouroussa Prefecture, Kankan, Guinea; 4 Direction Prefectorale de la Santé de Kouroussa, Kankan, Guinea

**Keywords:** Ebola, misconception, prevention, comprehensive knowledge

## Abstract

**Introduction:**

Guinea is the third hardest hit country in the region with 2,806 cases and 1,814 deaths as of January 11, 2015 after Sierra Leone and Liberia respectively. This KAP study was conducted in three sub-prefectures of Kouroussa in the Kankan region of Guinea from 15 December 2014 to 15 January 2015. It was conducted with the general objective of examining the knowledge, attitude and practice related to Ebola prevention and care among the public of Kouroussa Prefecture.

**Methods:**

A cross-sectional study design was employed to collect quantitative data to examine knowledge, attitude and practice related to Ebola. Structured questionnaire was administered by trained data collectors who were supervised by doctors and epidemiologists from WHO and Africa Union. Data were collected from 358 individuals (93% response rate) and analyzed in STATA 13 while tables and graphs are used to display results.

**Results:**

Over 96% of the respondents have ever heard about Ebola while only 76.2% believed the disease existed in Kouroussa. Avoiding physical contacts including hand shaking and contacts with body fluids, and early treatment of persons sick from Ebola were the two important prevention methods frequently mentioned (96.8% and 93.9%). Only 35.7% of respondents were found to have comprehensive knowledge about Ebola (composite of correctly accepting three methods of prevention (85%) and rejecting misconceptions (55.7%)).

**Conclusion:**

The high level of knowledge about modes of transmission and prevention methods has not positively affected the level of comprehensive knowledge about Ebola. In contrast, the prevailing high level of misconceptions surrounding Ebola was found to be responsible for a low comprehensive knowledge.

## Introduction

Ebola also known as Ebola Virus Disease (EVD) is a rare and deadly disease caused by infection with a virus of the family filoviridae, genus Ebola virus. Even though the natural reservoir remains unknown, evidences lead to conclude that the virus is animal-borne where bats are the most likely reservoir [1]. The current Ebola outbreak in West Africa is the largest and most complex outbreak in history since it was first discovered in 1976 in Congo. The first case was notified in March 2014 and it is believed that this outbreak started in Guinea and further spread to neighboring countries of Sierra Leone and Liberia [2]. Sierra Leone, Liberia and Guinea are the hardest hit countries by the outbreak while other countries including Nigeria, Mali, Senegal, Spain, United Kingdom and United States were also affected. As of 11 January 2015, a total of 21,261 cases and 8,414 deaths were reported from the three West African countries. Guinea is the third hardest hit country in the region with 2,806 cases and 1,814 deaths as of January 11, 2015 [3]. The WHO Patient database shows that a total of 19 cases have been reported so far from Kouroussa Prefecture during this outbreak where it claimed the lives of 11 people [4].

### Transmission of Ebola Virus Disease

According to the Centers of Disease Control and Prevention (CDC), the Ebola virus can spread through direct contacts with blood or body fluids of a person who is sick with Ebola, objects that have been contaminated with the virus, and infected fruit bats or primates (apes and monkeys). There is no evidence that Ebola is transmitted through the air, by water, or through mosquito bites [5]. In Knowledge, Attitude and Practice (KAP) study conducted in Sierra Leone in 2014, generally low level of knowledge was found regarding modes of transmission of EVD. Only 55% of respondents believed that physical contacts with known Ebola patient could transmit the disease while 32% and 43% believed Ebola could be transmitted through contact with blood and other body fluids. Preparing or eating bush meat was mentioned by 52% of the respondents to transmit the disease. On the other hand, about 30% of the respondents believe that EVD could be transmitted through air or mosquito bites [6].

### Prevention and control of Ebola Virus Disease

Careful practice of hygiene remains the mainstay to prevent transmission of EVD. Washing hands with soap and water or an alcohol based hand sanitizer and avoiding contacts with blood and body fluids are the main hygienic practices to prevent EVD. In addition, avoiding funeral or burial rituals that require handling the body of someone who has died from Ebola, and avoiding contact with bats and nonhuman primates or blood, fluids, and raw meat prepared from these animals are among the necessary steps to avoid contracting EVD [7]. Regarding the chances of surviving once caught the disease, evidences suggest that intensive treatment as early as possible in the course of illness would boost survival [8].

The KAP report from Sierra Leone shows that 87% of respondents agreed that Ebola could be prevented by avoiding handling blood or any other body fluids while 85% believed that avoiding funerals that require touching of the dead body could prevent Ebola. Similarly, 91% agreed that by immediately taking a suspect case to health facilities the spread of Ebola in the community could be reduced. The report also shows that significant proportion of the community reported misconceptions regarding prevention and treatment of Ebola. About 40% of the respondents believe that they can prevent Ebola by bathing in salty water while about 20% believe that spiritual healers can successfully treat the disease [6]. There is no KAP study related to Ebola conducted in Guinea and this piece is meant to fill the gap and to inform the behavior change communication strategies in the combat against Ebola.

### Methods

Study area: Kouroussa is a Prefecture located in the Kankan Region of Guinea, at about 600 kilometers east of Conakry, the Capital. The Prefecture is inhabited by an estimated population of 268,224. The study was specifically conducted in Kouroussa Commune, and Sanguiana and Babila Sub-Prefectures with populations of 39,611, 23,809 and 16,290 respectively. The study was limited to these three areas due to factors that include time and resource constraints and the fact that all the contacts to known Ebola cases lived only in these areas before or during the period of the study.

**Study period:** The study was conducted from 15 December 2014 to 15 January 2015.

**Study design:** a cross-sectional study design was employed to collect quantitative data to examine knowledge, attitude and practice related to Ebola.

**Sampling:** a generic formula of n=Z2.P(1-P)/d2 was used to determine the sample size (n) of 384 for this study where Z is a Z-score at 95% confidence level, P is a population estimate (50%) and d is 5% margin of error. Population proportion sampling calculation was then applied to allocate sample sizes to sub-prefectures. Accordingly, sample sizes of 191, 115, and 78 were allocated to Kouroussa Commune, Sanguiana and Babila respectively.

**Selection of study participants:** households were selected from each village using systematic random sampling technique where estimated number of households in each sub-Prefecture was used to determine the intervals. Once a household was identified one person per household was selected with preference given to head of household downwards the hierarchy to any one at home during the survey with the minimum age of inclusion being 18 years.

**Data collection and analysis:** structured questionnaire was administered by trained data collectors who were supervised by doctors and epidemiologists from WHO and Africa Union. Data were analyzed in STATA 13 while tables and graphs are used to display results.

**Ethical considerations:** approval was obtained from Director of Kouroussa Direction Prefecturale De la Sante, Prefectural Ebola Coordination Body, and Prefectural Council. Informed consents were obtained from individual respondents during the survey.

**Limitations of the study:** the study was limited to Kouroussa commune and two sub-prefectures due to the factors mentioned above and this may affect generalizability of the findings to the entire prefecture. The study team, however, does not expect significant variability across the prefecture and as a result limitation to cover wider area may not seriously affect the study.

## Results

The results of the survey are presented in this section in tables or graphs followed by brief interpretations.

### Response rate and characteristics of respondents

Data were collected from 93% of the initially intended respondents with the minimum and maximum being 91%and 99% in Kouroussa and Babila respectively (Table 1). About 48% of the respondents were from Kouroussa commune while Babila contributed only 21.5% of the response. About 58% of the respondents were male while the remaining was constituted by their female counter part.

**Table 1:** Ebola KAP survey response rate, Kouroussa, 2015

Sub-Prefecture	Population	Proportion of Population	Sample Size	Proportion of Sample	Response rate	
					Responded	Percentage
Kouroussa	39,611	50%	191	50%	173	91%
Sanguiana	23,809	30%	115	30%	108	94%
Babila	16,290	20%	78	20%	77	99%
**Total**	**79,710**	**100%**	**384**	**100%**	**358**	**93%**

### Community awareness about Ebola

Table 2 shows that reasonable proportion of the respondents (96.4%) have ever heard about Ebola while below half had known anyone who had survived Ebola. About 98% of those who have ever heard about Ebola believed that Ebola existed in Guinea during the survey period while only 76% believed that it existed in the prefecture.

**Table 2:** Community Awareness of Ebola by socio-demographic characteristics, Kouroussa, 2015

	Have heard of EVD	Believe EVD exists in Guinea	Believe EVD exists in Kouroussa	Have heard of EVD survivors	Know number to call	Total respondents
**Sub-prefecture**						
Kouroussa	98.8	98.3	87.1	41.5	52.6	173
Sanguiana	89.8	97.9	36.4	40.3	56.7	108
Babila	100	98.7	88.7	70.1	85.7	77
**Sex**						
Male	96.1	99.0	78.7	50.3	67.3	207
Female	96.7	97.3	75.3	49.3	52.7	151
**Age**						
18-24	97.8	97.7	75.0	47.7	79.6	45
25 and above	96.2	98.3	76.4	49.5	58.5	313
**Marital status**						
Married	95.7	98.0	74.1	49.4	49.4	258
Single	98.6	100	89.9	53.6	53.6	70
Divorced	100	83.3	50.0	33.3	33.3	6
Widowed	95.5	100	61.9	42.9	42.9	22
Separated	100	100	100	0.0	0.0	2
**Education**						
No formal education	92.7	97.0	70.1	39.6	39.6	177
Primary	100	100	71.2	46.2	46.2	52
Secondary	100	98.8	84.7	62.4	62.4	85
College/University	100	100	87.8	65.6	65.9	41
**Occupation**						
Trading	99.0	97.9	82.1	48.4	48.4	96
Farmer	89.9	97.5	63.8	40.0	40.0	89
Fisher	90.9	100	70.0	30.0	30.0	11
Employee (all forms)	100	100	83.3	69.7	69.7	66
No job/Unemployed	97.5	97.4	77.9	50.7	50.7	79
Other	100	100	70.6	23.5	23.5	17
**Total**	**96.4**	**98.3**	**76.2**	**49.3**	**61.2**	**358**

### Causes of Ebola

**Figure 1:**
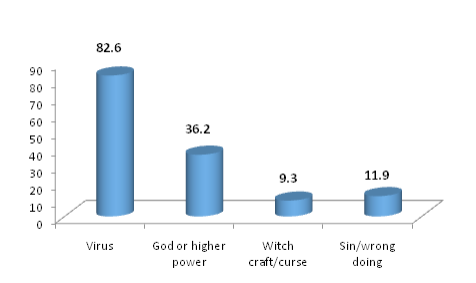
Summary of responses on causes of Ebola, Kouroussa, 2015

Figure 1 shows that over 82% of the respondents believe that Ebola is caused by virus whereas significant proportion (36.2%) believe that it is brought to humans by God or other higher power. About 12% believe that Ebola happens to someone due to sins or wrong doings. The beliefs were found to have varied across socio-demographic characteristics.

### Modes of Transmission of EVD

Table 3 presents percentage of responses on the mode of transmission of EVD. About 90% of the respondents believed that any physical contact that include hand shaking could transmit EVD while about 89% believe that handling blood or body fluids of a person who is infected with Ebola virus could transmit the disease. Preparing or eating bush meat and wild fruits (69% and 58.8% respectively) were also well recognized by the respondents to transmit EVD. Education appears to be positively associated with the level of knowledge of modes of transmission of EVD except for physical contacts and contacts with body fluids. The results exhibit little variations in the level of knowledge about the modes of transmission across various socio-demographic characteristics.

**Table 3:** Percentage of responses about mode of transmission of Ebola, Kouroussa, 2015

	Touching infected person (Alive/dead)	Touching body fluids of symptomatic case	Eating or preparing bush meat	Eating wild fruits	Sperm of infected person	Breast milk of infected mother	
**Sub-prefecture**							
Kouroussa	92.6	90.1	68.4	61.4	66.7	68.4	173
Sanguiana	91.8	90.7	58.8	41.2	69.1	63.9	108
Babila	85.4	83.1	83.1	75.3	81.8	84.4	77
**Sex**							
Male	89.5	87.4	69.4	63.3	73.9	75.4	207
Female	91.8	90.4	68.5	52.7	66.4	64.4	151
**Age**							
18-24	88.4	95.5	81.8	77.3	75.0	72.7	45
25 and above	86.1	87.7	67.1	56.2	70.1	70.4	313
**Marital status**							
Married	87.5	86.6	67.2	58.7	69.2	70.9	258
Single	97.1	91.3	72.5	65.2	79.7	71.0	70
Divorced	100	100	83.3	50.0	s50.0	50.0	6
Widowed	100	100	71.4	38.1	61.9	71.4	22
Separated	100	100	100	100	100	100	2
**Education**							
No formal education	90.2	90.9	66.5	53.7	68.3	72.0	177
Primary	86.5	84.6	65.4	59.6	65.4	63.5	52
Secondary	92.9	89.4	72.9	63.5	72.9	71.8	85
College/University	95.1	87.8	75.6	68.3	82.9	73.2	41
**Occupation**							
Trading	84.2	82.1	71.6	65.3	81.1	73.7	96
Farmer	87.5	90.0	63.8	55.0	67.5	78.8	89
Fisher	90.0	80.0	80.0	70.0	80.0	90.0	11
Employee (all forms)	97.0	90.9	74.2	59.1	68.2	68.2	66
No job/ Unemployed	93.5	93.5	66.2	57.1	72.7	64.9	79
Other	100	94.1	64.7	41.2	23.5	41.2	17
**Total**	**90.4**	**88.7**	**69.0**	**58.8**	**70.7**	**70.7**	**345**

### Attitudes and perceptions towards EVD prevention

Avoiding physical contacts with a person with symptoms of Ebola whether alive or dead is a method of prevention that is well recognized by the respondents (96.8%). About 94% of the respondents also believe that taking a person who sick from Ebola to health facility as soon as possible would reduce the risk of spreading the disease in the community. Pretty reasonable proportion, however, would either tend to hide the sick or do nothing about it if someone happens to be sick from Ebola, 30.7% and 30.4% respectively (Table 4).

**Table 4:** Attitudes and perceptions of respondents towards prevention of Ebola, Kouroussa, 2015

	Avoid physical contact (alive/dead)	Taking sick person immediately to health facility reduces risk of transmission	Calling health worker immediately	Hiding the sick so that people are not infected	There is nothing someone can do to prevent	Total
**Sub-prefecture**						
Kouroussa	97.0	92.3	92.3	34.5	47.6	168
Sanguiana	93.8	91.8	84.5	26.8	9.3	97
Babila	100.0	100.0	93.5	27.3	19.5	77
**Sex**						
Male	96.5	94.9	91.9	34.0	29.4	190
Female	97.2	92.4	88.3	26.2	31.7	141
**Age**						
18-24	93.0	93.0	93.0	23.3	25.6	40
25 and above	97.3	94.0	90.0	31.8	31.1	291
**Marital status**						
Married	96.3	93.9	91.0	34.7	31.8	245
Single	97.1	92.7	89.7	23.5	25.0	68
Divorced	100.0	83.3	66.7	0.0	33.3	6
Widowed	100.0	100.0	90.5	19.1	33.3	21
Separated	100.0	100.0	100.0	0.0	0.0	2
**Education**						
No formal education	95.7	95.1	86.5	31.9	38.7	163
Primary	96.2	88.5	94.2	34.6	32.7	52
Secondary	98.8	94.0	92.8	33.7	19.3	83
College/University	97.6	95.1	95.1	14.6	14.6	41
**Total**	**96.8**	**93.9**	**90.4**	**30.7**	**30.4**	**342**

### Misconceptions surrounding EVD

**Figure 2:**
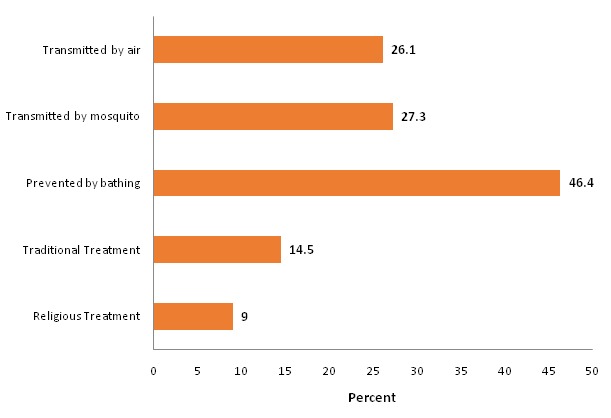
Percentages of misconceptions about transmission, prevention and treatment of Ebola, Kouroussa, 2015

Figure 2 presents percentage of responses that were not in line with scientific evidences and therefore recognized as misconceptions about EVD. About 46% of respondents believe that Ebola could be prevented by bathing in salty water while 26% and 27% of respondents believe that Ebola could be successfully treated by traditional and religious healers respectively.

### Comprehensive knowledge about EVD

Comprehensive knowledge in this context is defined as accepting three main methods of prevention and rejecting three major misconceptions. The three methods of transmission that are critical to prevent EVD include avoiding physical contacts with Ebola cases whether alive or dead, avoiding handling blood or body fluids of a person sick from Ebola, and washing hands with soap and water or chlorinated water or alcohol based hand sanitizer. On the other hand, the three major misconceptions that need to be rejected include perceptions that EVD is transmitted by air or by mosquito, and that EVD could be prevented by bathing in salty water. The following table presents percentage of respondents that accept three methods of prevention and three major misconceptions about Ebola.

Results in Table 5 show that significant proportions of respondents (85%) have correctly identified the three critical methods of preventing Ebola. However, only 56% of the respondents rejected the misconceptions that are detrimental in the fight against Ebola. The comprehensive knowledge about Ebola (35.7%) has been seriously affected by the interplay between accepting appropriate methods of prevention and rejecting misconceptions. While there is little variation across the socio-demographic characteristics education appears to be positively associated with comprehensive knowledge about Ebola.

**Table 5:** Percentage of respondents who correctly identified prevention methods and rejected misconceptions, Kouroussa, 2015

	Accepts three main means of prevention		Rejects three misconceptions		Has comprehensive knowledge (Accepts three main means of prevention and Rejects three misconceptions)	
	Number	Percent	Number	Percent	Number	Percent
**Sub-prefecture**						
Kouroussa	149	87.1	113	66.1	43	25.2
Sanguiana	78	80.4	29	26.9	56	57.8
Babila	69	89.6	51	66.2	24	31.2
**Sex**						
Male	174	87.4	123	61.8	63	31.7
Female	122	83.6	70	48.0	60	41.1
**Age**						
18-24	38	86.4	19	43.2	21	47.7
25 and above	258	85.7	174	57.8	102	33.9
**Marital status**						
Married	211	85.4	146	59.1	81	32.8
Single	59	85.5	37	53.6	25	36.2
Divorced	5	83.3	1	16.7	5	83.3
Widowed	19	90.5	8	38.1	11	52.4
Separated	2	100.0	1	50.0	1	50.0
**Education**						
No formal education	138	84.2	89	54.3	57	34.8
Primary	45	86.5	35	67.3	15	28.9
Secondary	72	84.7	47	55.3	29	34.1
College/University	38	92.7	21	51.2	20	48.8
**Occupation**						
Trading	82	86.3	69	72.6	22	23.2
Farmer	68	85.0	45	56.3	28	35.0
Fisher	9	90.0	7	70.0	3	30.0
Employee (all forms)	57	86.4	32	48.5	28	42.4
No job/ Unemployed	67	87.0	38	49.4	31	40.3
Other	13	76.5	2	11.8	11	64.7
**Total**	**296**	**85.0**	**193**	**55.9**	**123**	**35.7**

## Discussion

The percentage of respondents who have reported to have ever heard about Ebola (96.4%) was significantly lower than the finding from a neighboring country Sierra Leone (100%) [6]. Even though the belief that EVD exists in Guinea was as high as 98.3%, the belief that it existed in Kouroussa was very low (76.2%). In the contrary, reports reveal that 19 people were sick from Ebola in the prefecture where it claimed lives of 11 people [4]. On the top, it would be enormously unconvincing to fail to hear about the occurrence of a rare and deadly disease like Ebola when it was already in the local and international media. The disagreement between these facts, therefore, shows that there is certain degree of disbelief that Ebola is a risk.

Knowledge about someone who has survived EVD is only at 49.3% and this suggests that using survivors in awareness raising activities would help in combating the disease better. This is important because chances of survival are dependent on many factors among which early treatment is a key and is a behavior the community needs to adopt [8]. Despite high level of knowledge that virus causes EVD (82.6%), significant proportion (36.2%) believes that Ebola is caused by God or other higher powers that are beyond human control. This is much higher than the finding in a neighboring country Sierra Leone (1.7%) [6]. This finding may suggest that an intervention needs to be in place as prevention is mainly dependent on the knowledge of the causes. The attitude that someone should be taken to a health facility as soon as he/she falls sick (93.9%) is higher than the finding in Sierra Leone (91.3%) and needs strengthening.

The comprehensive knowledge level of the respondents about EVD was very low (35.7%) in spite of the fact that 85% of them correctly identified the three main methods of preventing EVD (Table 5). This is close to the finding from Sierra Leone where it was at 38.8% [6]. The comprehensive knowledge about Ebola was affected by the interplay between accepting appropriate methods of prevention and rejecting misconceptions. The belief that bathing in salty water would prevent Ebola (46.4%) (Figure 2) was the prominent misconception that skewed the level of comprehensive knowledge about EVD.

## Conclusion

The apparently high proportion (96.4%) of respondents that have ever heard about Ebola is not sufficient to effectively control and prevent the disease. Given the nature of the disease and prevalence of information in the local and international media, the deficit of 3.6% of hearing about Ebola remains unacceptable. To make things worse, only 76.2% believe that the disease exists in Kouroussa, a prefecture where 19 were sick from the disease and 11 have already died.

On the other hand, the significantly high level of attitude and perceptions towards prevention (avoid physical contacts=96.8%, early treatment of cases=93.9%) is not commensurate with the level of correctly identifying the three main prevention methods (85%) and rejecting misconceptions (55.9%). The resultant of the interplay between the failure to adopt appropriate prevention methods and failure to reject misconceptions has left the comprehensive knowledge about Ebola in vain. Continuous high level advocacy and awareness raising activities need to be put into operation to refute the seemingly prevalent denial (only 96.4 to have heard of Ebola, and only 76.2% to have believed it existed in Kousoussa) of existence of EVD.
